# IRE1α-XBP1 is a novel branch in the transcriptional regulation of *Ucp1* in brown adipocytes

**DOI:** 10.1038/srep16580

**Published:** 2015-11-16

**Authors:** Rie Asada, Soshi Kanemoto, Koji Matsuhisa, Kenta Hino, Min Cui, Xiang Cui, Masayuki Kaneko, Kazunori Imaizumi

**Affiliations:** 1 Department of Biochemistry, Institute of Biomedical & Health Science, Hiroshima University, 1-2-3 Kasumi, Minami-ku, Hiroshima 734-8553, Japan

## Abstract

The unfolded protein response (UPR) not only resolves endoplasmic reticulum (ER) stress, but also regulates cellular physiological functions. In this study, we first linked the UPR to the physiological roles of brown adipose tissue (BAT). BAT is one of the tissues that control energy homeostasis in the body. Brown adipocytes are able to dissipate energy in the form of heat owing to their mitochondrial protein, uncoupling protein 1 (UCP1). We found that one of the UPR branches, the IRE1α-XBP1 pathway, was activated during the transcriptional induction of *Ucp1*. Inhibiting the IRE1α-XBP1 pathway reduced the induction of *Ucp1* expression. However, the activation of the IRE1α-XBP1 pathway by ER stress never upregulated *Ucp1*. On the other hand, the activation of protein kinase A (PKA) induced *Ucp1* transcription through the activation of IRE1α-XBP1. The inhibition of PKA abrogated the activation of IRE1α-XBP1 pathway, while the inhibition of a p38 mitogen activated protein kinase (p38 MAPK), which is one of the downstream molecules of PKA, never suppressed the activation of IRE1α-XBP1 pathway. These data indicate that PKA-dependent IRE1α-XBP1 activation is crucial for the transcriptional induction of *Ucp1* in brown adipocytes, and they demonstrate a novel, ER stress -independent role of the UPR during thermogenesis.

The endoplasmic reticulum (ER) is a central cellular organelle that is responsible for the folding and post-translational modification of transmembrane and secretory proteins, as well as lipid synthesis. Various genetic and environmental insults lead to the accumulation of unfolded or misfolded proteins in the ER lumen. These conditions, collectively termed ER stress, have the potential to induce cellular damage. Excessive ER stress ultimately leads to apoptotic cell death. Eukaryotic cells are equipped with a system, known as the unfolded protein response (UPR), that prevents the cellular damage and death induced by ER stress^1^,^2^. In mammalian cells, three major ER stress transducers have been identified: PKR-like ER kinase (PERK)^3^, inositol-requiring enzyme 1 (IRE1)^4^,^5^, and activating factor 6 (ATF6)^6^. In response to ER stress, these transducers promote three distinct physiological responses: 1) translational attenuation to decrease the demands made on the organelle^7^, 2) the transcriptional induction of genes encoding ER-resident chaperones to facilitate protein folding^6^,^8^, and 3) ER-associated degradation (ERAD) to degrade unfolded or misfolded proteins in the ER lumen^9^,^10^.

During the last decade, a number of studies have indicated that the UPR has crosstalk with other signals and participates in regulating cellular physiological functions. It has been reported that UPR signals from the ER are indispensable for the differentiation of plasma cells^11^, astrocytes^12^, osteoblasts^13^,^14^, chondrocytes^15^, and goblet cells^16^. Recently, it was also demonstrated that the UPR is involved in cellular proliferation or tumorigenesis^17^. Interestingly, it was revealed that changes in the lipid composition of the ER membrane bilayer can activate ER stress transducers, leading to the regulation of lipid metabolism^18^,^19^. Thus, the UPR has diverse roles beyond getting rid of unfolded or misfolded proteins from the ER lumen.

Of the three major ER stress transducers, IRE1α is the most evolutionarily conserved. In addition to its kinase domain, IRE1α also has an endo-nuclease domain^4^,^5^. Activation of IRE1α via dimerization and *trans*-autophosphorylation causes the unconventional splicing of X-box binding protein 1 (*Xbp1*) mRNA by its nuclease activity^20^. Spliced *Xbp1* (s*Xbp1*)-encoded proteins can translocate into the nucleus and induce the expression of ER-resident chaperones and ERAD components^21^,^22^. It was suggested that this IRE1α-XBP1 pathway directly influences lipid metabolism by regulating the expression of lipogenic genes such as *Dgat2*, *Scd1*, and *Acc2*^23^. Furthermore, obesity impairs sXBP1 activity in the liver, and overexpression of sXBP1 in obese or diabetic mice improves the blood glucose level to euglycemia^24^,^25^,^26^. Collectively, it is believed that sXBP1 plays a crucial role in the regulation of lipid and glucose homeostasis, as well as in the pathogenesis of metabolic diseases, including obesity and diabetes.

Brown adipose tissue (BAT) is a key tissue that controls the energy balance of whole body. Excessive energy is dissipated in the form of heat by brown adipocytes specialized for thermogenesis^27^,^28^,^29^. BAT plays important roles in protecting neonates and small mammals, such as mice, against cold^30^. The unique metabolic properties of brown adipocytes result from their densely packed mitochondria containing uncoupling protein 1 (UCP1) in their inner membrane. UCP1 is almost exclusively expressed in brown adipocytes, and it is a proton transporter that allows protons to leak into the mitochondrial matrix without ATP synthesis, which leads to thermogenesis^31^. The transcription of *Ucp1* is regulated by several transcriptional factors and co-regulators, including activating transcription factor 2 (ATF2), cyclic-AMP (cAMP) responsive element binding protein (CREB), and peroxisome proliferative activated receptor gamma coactivator 1 alpha (PGC1α)^28^,^32^. Because this energy consumption system in BAT is attractive for counteracting obesity and its related metabolic diseases^33^, it is important to understand the mechanisms that increase *Ucp1* expression. In this study, we first uncovered a physiological role of UPR in BAT, and we demonstrated that the IRE1α-XBP1 pathway is activated in a PKA-dependent manner and plays a crucial role in the transcriptional induction of *Ucp1* in brown adipocytes.

## Results

### The UPR is activated during the transcriptional induction of *Ucp1* in brown adipocytes

**Figure 1:**
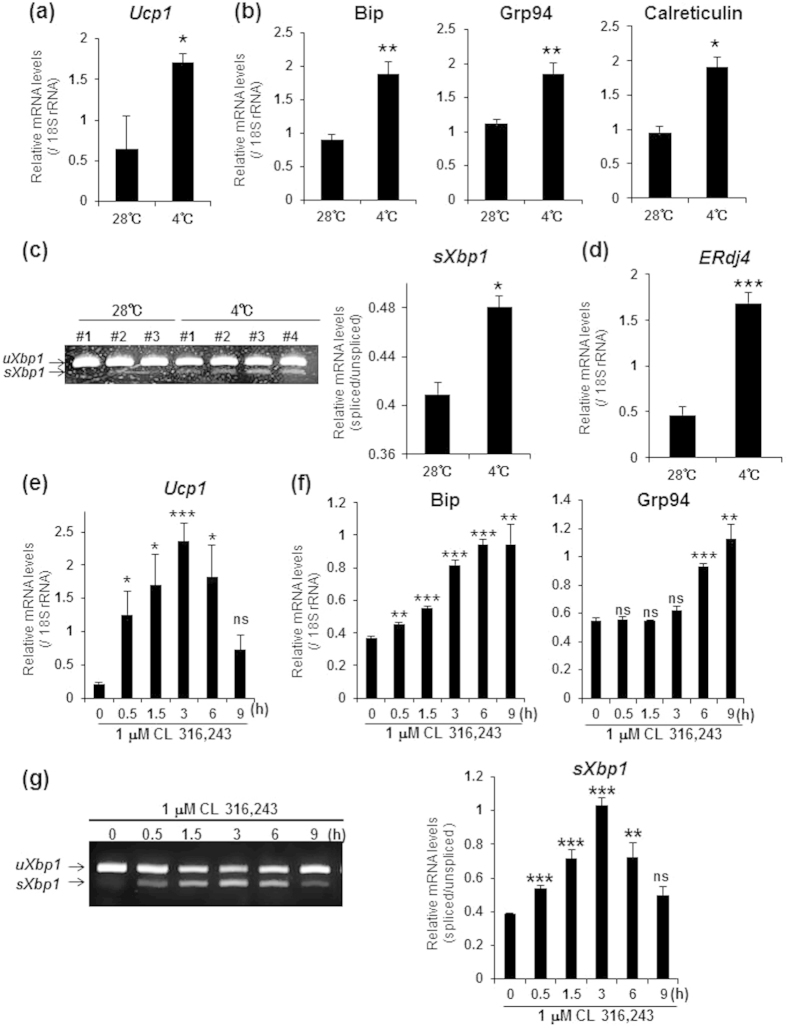
The UPR-related genes were upregulated during the transcriptional induction of *Ucp1* in brown adipocytes. (**a**,**b**) Real-time PCR analysis of *Ucp1* (**a**), and UPR-related genes (**b**) in BAT exposed to cold (4 °C) for 24 h, or in control (28 °C) BAT. (**c**) RT-PCR analysis of *Xbp1* in BAT exposed to cold (left panel). *uXbp1* and *sXbp1* indicate unspliced and spliced forms of *Xbp1*, respectively. Graph on right shows the quantification of *Xbp1* splicing levels. (**d**) Real-time analysis of a target gene of sXBP1, *Erdj4* in BAT exposed to cold. Differences between control and cold exposure were analyzed by Student’s t-test. Data are mean ± S.D. (control : n = 3, cold: n = 4) *P < 0.05, **P < 0.01, ***P < 0.001. (**e**,**f**) Real-time PCR analysis of *Ucp1* (**e**), and UPR-related genes (**f**) in brown adipocytes treated with 1 μM CL 316,243 for indicated time periods. (**g**) RT-PCR analysis of *Xbp1* in brown adipocytes treated with CL 316,243 (left panel). Graph on right shows the quantification of *Xbp1* splicing levels. Note that splicing levels were increased in accordance with the expression pattern of *Ucp1*. Differences with and without treatment were analyzed by Student’s t-test. Data are mean ± S.D. (n = 4), *P < 0.05, **P < 0.01, ***P < 0.001, ns: not significant.

To examine whether UPR is activated in response to stimuli leading to thermogenesis in brown adipocytes, we exposed C57BL/6 mice to cold (4 °C) for 24 h, isolated mRNA from BAT, and then measured the expression levels of UPR-related genes by real-time PCR analysis. It is well known that cold exposure promotes the transcription of *Ucp*1 for thermogenesis^27^,^28^. In our study, a significant elevation of *Ucp1* mRNA levels was observed in BAT of mice exposed to cold (4 °C) for 24 h compared with control mice that were kept at 28 °C (Fig. 1a). The expression of the ER-resident chaperones Bip, Grp94, and Calreticulin was significantly upregulated by cold exposure (Fig. 1b). According to western -blotting (WB) analysis, the levels of Bip proteins significantly increased, and GRP94 and a component of the UPR signal, ATF4, tended to increase after cold exposure (Supplementary Fig. 1). RT-PCR analysis showed an increase in the level of *sXbp*1 mRNA in BAT exposed to cold (Fig. 1c). Additionally, the mRNA level of *ERdj4*, which is a target gene of sXBP1, also increased (Fig. 1d). Cold exposure promotes not only thermogenesis, but also various physiological responses in BAT, such as angiogenesis^34^. Therefore, we next asked whether the increase in the expression of UPR-related genes was due to thermogenesis in brown adipocytes in response to cold. Norepinephrine is a major activator of brown adipocytes that promotes *Ucp1* expression by binding to the β_3_ adrenergic receptor (β_3_-AR), which is expressed mainly in adipose tissue. Treatment with a β_3_-AR agonist such as CL 316,243 can mimic cold exposure in brown adipocytes^27^. Indeed, subcutaneous injection of CL 316,243 (1 mg/kg) induced a significant increase in *Ucp1* mRNA levels in BAT (Supplementary Fig. 2a). The expression of the ER-resident chaperones, Bip, Grp94, and Calreticulin, as well as *sXbp1* mRNA, was increased by CL 316,243 (Supplementary Fig. 2b,c). Treatment of primary brown adipocytes with 1 μM CL 316,243 transiently elevated *Ucp1* mRNA levels, which peaked 3 h after treatment (Fig. 1e). The expression of Bip and Grp94 was also upregulated by treatment with CL 316,243, and this increase in expression remained until 9 h after treatment (Fig. 1f). On the other hand, splicing of *Xbp1* mRNA increased in accordance with the expression pattern of *Ucp1* (Fig. 1g), implying that sXBP1 acts during an early phase of the physiological response of brown adipocytes to norepinephrine, although ER-resident chaperones may not be needed until a later phase.

### The IRE1α-XBP1 pathway is predominantly activated during the induction of *Ucp1* transcription

**Figure 2:**
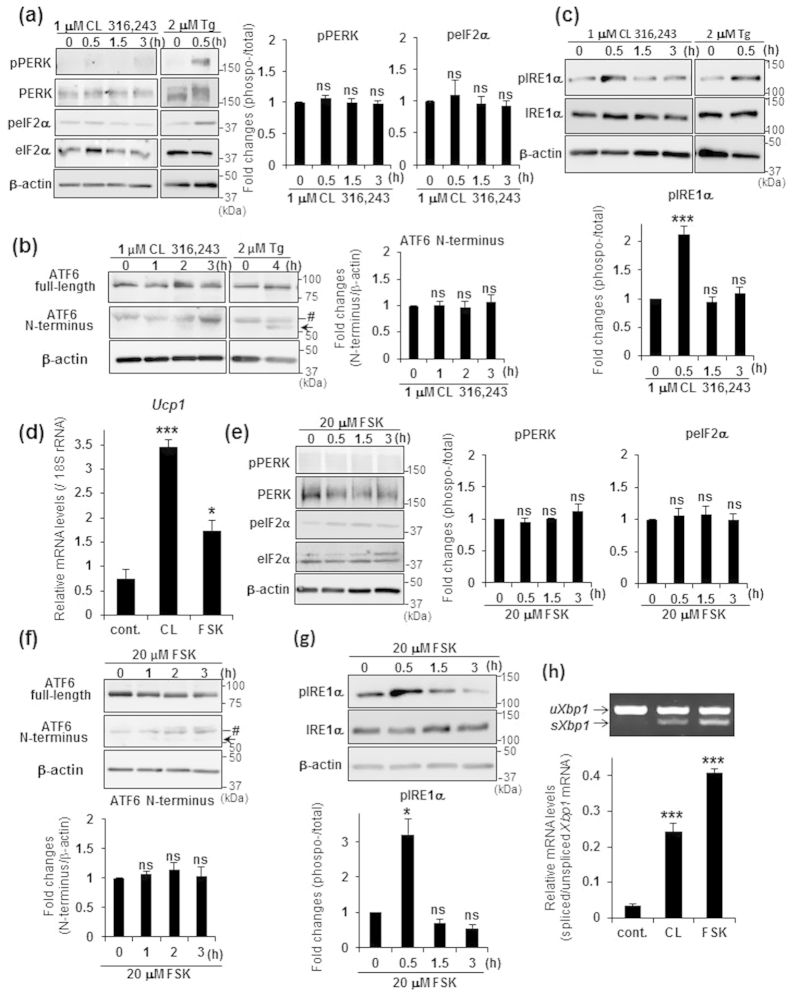
IRE1α was predominantly activated by treatment with β_3_-AR agonist or forskolin. (**a**–**c**) WB analysis of PERK, eIF2α (**a**), ATF6 (**b**), and IRE1α (**c**) in brown adipocytes treated with either 1 μM CL 316,243 or 2 μM Tg for indicated time periods. Black arrow and Mark (#) indicate ATF6 N-terminus and non-specific detections, respectively. β-actin was used as a loading control. Graphs show the ratio of phosphorylated to total PERK, eIF2α, or IRE1α, and the amount of ATF6 N-terminus. Note that phosphorylation of IRE1α was significantly increased by treatment with CL 316,243. Differences with and without treatment were analyzed by Student’s t-test. Data are mean ± S.D. (n = 4), ***P < 0.001, ns: not significant. (**d**) Real-time PCR analysis of *Ucp1* in brown adipocytes treated with either 1 μM CL 316,243 (CL) or 20 μM forskolin (FSK) for 3 h. Differences with and without treatment were analyzed by Student’s t-test. Data are mean ± S.D. (n = 3), *P < 0.05, ***P < 0.001. cont.: control. (**e**–**g**) WB analysis of PERK, eIF2α (**e**), ATF6 (**f**), and IRE1α (**g**) in brown adipocytes treated with 20 μM FSK for indicated time periods. Black arrow and Mark (#) indicate ATF6 N-terminus and non-specific detections, respectively. β-actin was used as a loading control. Graphs show the ratio of phosphorylated to total PERK, eIF2α, or IRE1α, and the amount of ATF6 N-terminus. Differences with and without treatment were analyzed by Student’s t-test. Data are mean ± S.D. (n = 4), *P < 0.05, ns: not significant. (**h**) RT-PCR analysis of *Xbp1* in brown adipocytes treated with either 1 μM CL 316,243 (CL) or 20 μM forskolin (FSK) for 3 h (upper panel). Lower graph is the quantification of *Xbp1* splicing levels. Differences with and without treatment were analyzed by Student’s t-test. Data are mean ± S.D. (n = 3), ***P < 0.001. cont.: control.

Next, we examined which branch in the UPR is important for the transcriptional induction of *Ucp1* in brown adipocytes. Because the largest increase in *Ucp1* mRNA was observed 3 h after the treatment of primary brown adipocytes with CL 316,243, we conducted WB analysis to measure a change in the amount of phosphorylated PERK, eIF2α, and IRE1α, or N-terminus of ATF6 until 3 h after treatment. As shown in Fig. 2a, treatment with 1 μM CL 316,243 never induced the phosphorylation of PERK or its substrate eIF2α, whereas treatment with the ER stressor thapsigargin (Tg) substantially increased the phosphorylation of both proteins in primary brown adipocytes. The cleaved fragment of ATF6 (ATF6 N-terminus) did not increase after treatment with CL 316,243 (Fig. 2b). In contrast, the phosphorylation of IRE1α significantly increased 30 min after treatment with CL 316,243 (Fig. 2c). These data indicated that IRE1α is predominantly activated downstream of the β_3_-AR pathway. It is not known the reason why the phosphorylation of IRE1α swiftly went back to the basal level, while *sXbp1* mRNA was detected until 6 h after CL 316,243 treatment. We speculated two possibilities: 1) Because our antibody detects only phosphorylation of Ser 724 of IRE1α, the other phosphorylation sites in multiple phosphorylation sites of IRE1α could be phosphorylated with CL 316,243 treatment time. 2) Because the stability of *sXbp1* mRNA is high, *sXbp1* mRNA could escape from rapid degradation.

Binding of norepinephrine to the β_3_-AR activates adenylyl cyclase, leading to an increase in the concentration of cAMP and an increase in PKA activity. To determine whether the β_3_-AR pathway affects the activation of IRE1α, we treated primary brown adipocytes with forskolin (FSK), an activator of adenylyl cyclase, which thereby results in an increase in intracellular cAMP. *Ucp1* was upregulated by treatment with 20 μM FSK, although the level of upregulation was less than that observed following CL 316,243 treatment (Fig. 2d). WB analysis showed that PERK and eIF2α were never phosphorylated after treatment with FSK (Fig. 2e). ATF6 N-terminus also did not increase after treatment with FSK (Fig. 2f). These results suggested that the PERK and ATF6 branches do not play crucial roles in the transcriptional induction of *Ucp1*. Interestingly, the phosphorylation of IRE1α and the splicing of *Xbp1* mRNA were enhanced after treatment with FSK compared with the CL 316,243 treatment (Fig. 2g,h), indicating that the activation of the IRE1α-XBP1 pathway is the most important of the three UPR branches in the induction of *Ucp1* transcription by the β_3_-AR pathway.

### The IRE1α-XBP1 pathway is essential for the transcriptional induction of *Ucp1*

**Figure 3:**
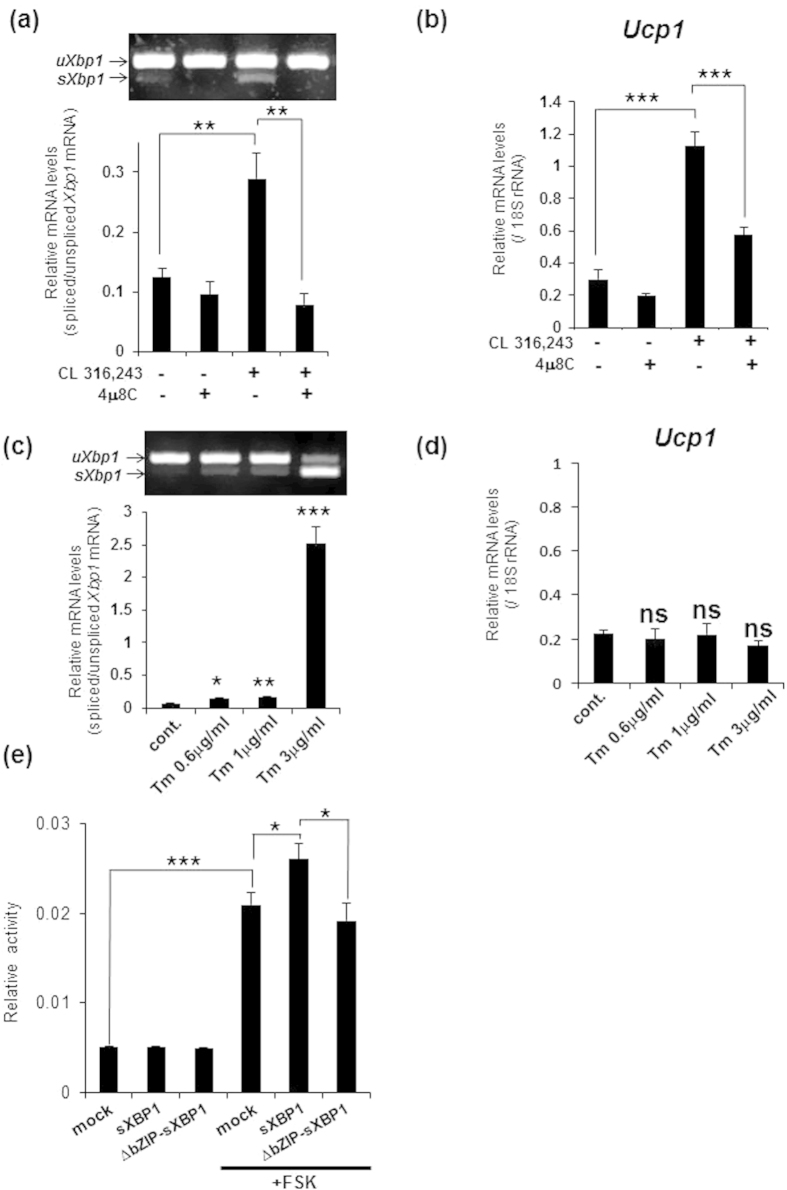
The IRE1α-XBP1 pathway plays a crucial role in the transcriptional induction of *Ucp1*. (**a**) RT-PCR analysis of *Xbp1* in brown adipocytes that were pre-treated with 30 μM 4 μ8 C for 30 min and then stimulated with 1 μM CL 316,243 for 3 h (upper panel). Lower graph is the quantification of *Xbp1* splicing levels. (**b**) Real-time PCR analysis of *Ucp1* in brown adipocytes treated with 4 μ8 C and CL 316,243 described as (**a**). Note that treatment with 4 μ8 C significantly decreased *Ucp1* expression induced by CL 316,243. Data are mean ± S.D. (n = 5), **P < 0.01, ***P < 0.001. (**c**) RT-PCR analysis of *Xbp1* in brown adipocytes that were treated for 3 h with tunicamycin (Tm) at the indicated concentrations (upper panel). Lower graph shows the quantification of *Xbp1* splicing levels. (**d**) Real-time PCR analysis of *Ucp1* in brown adipocytes treated with Tm described as (**c**). Differences with and without treatment were analyzed by Student’s t-test. Data are mean ± S.D. (n = 3), *P < 0.05, **P < 0.01, ***P < 0.001, ns: not significant, cont.: control. (**e**) Luciferase assay using C3H10T1/2 cells that were transfected with vectors expressing sXBP1 or ΔbZIP-sXBP1, and then treated with 20 μM forskolin (FSK) for 8 h. Note that the increase in the reporter activity by sXBP1 expression and FSK treatment was higher than the activity by FSK treatment alone. Data are mean ± S.D. (n = 6), *P < 0.05, ***P < 0.001.

To determine whether the IRE1α-XBP1 pathway is mainly involved in the transcriptional induction of *Ucp1*, we first attempted to investigate the effects of the knockdown of *Xbp1* in brown adipocytes using lentiviral vectors, because primary mature brown adipocytes do not proliferate. However, we could not detect green fluorescent protein (GFP) fluorescence in primary mature brown adipocytes infected with lentiviruses expressing GFP, indicating that the knockdown of *Xbp1* in mature brown adipocytes is technically difficult. Instead of gene silencing experiments, we treated primary brown adipocytes with 30 μM 4 μ8 C. 4 μ8 C inhibits the nuclease activity of IRE1α by directly binding to its active site without affecting its kinase activity or its overall dimerization or oligomerization states^35^. Using this compound, we successfully depleted the splicing of *Xbp1* mRNA induced by the β_3_-AR pathway (Fig. 3a). The quantification of *Ucp1* mRNA levels demonstrated a significant decrease after treatment with 4 μ8 C compared with treatment with CL 316,243 (Fig. 3b). Treatment with 4 μ8 C also inhibited the splicing of *Xbp1* mRNA and the upregulation of *Ucp1* expression following FSK treatment (Supplementary Fig. 3a,b), suggesting that the IRE1α-XBP1 pathway plays a crucial role in the transcriptional induction of *Ucp1*. Because ER stress activates the IREα-XBP1 pathway, we next examined whether ER stress is able to induce the transcription of *Ucp1*. Primary brown adipocytes were treated with various concentrations of tunicamycin (Tm), which blocks N-linked glycosylation and causes ER stress. Tm treatment caused significant splicing of *Xbp1* mRNA (Fig. 3c), but never induced *Ucp1* transcription (Fig. 3d). Therefore, the activation of the IRE1α-XBP1 pathway by the β_3_-AR pathway, which leads to the transcriptional induction of *Ucp1*, could occur independently of the canonical UPR that is induced by ER stress.

Next, we tested whether sXBP1 directly promotes the transcription of *Ucp1* because sXBP1 is a transcription factor that includes a basic leucine zipper (bZIP) DNA binding domain. It is well known that sXBP1 binds to the *cis*-acting ER stress response element (ERSE, CCAAT-N_9_-CCACG or ERSEII, ATTGG-N-CCACG^36^), and the mammalian unfolded protein response element (UPRE, TGACGTGG/A^22^) (underlined sequences indicate core sequences). A search using the murine genome database (Ensembl genome browser GRCm38) for these binding sequences in the promoter region (up to –4 kb upstream) of *Ucp1* identified several UPRE and ERSE core sequences (Supplementary Fig. 4a). Especially, we found two UPRE and one ERSE core sequences within 1 -kb of *Ucp1*, implying that sXBP1 directly binds these elements to promote *Ucp1* transcription. To verify this possibility, we performed luciferase assay using a reporter plasmid that contained a –3.8-kb promoter of murine *Ucp1*^37^. We transfected the reporter plasmid into C3H10T1/2 cells, which is a murine mesenchymal stem cell line that has been used to examine *Ucp1* promoter activity^37^. We confirmed that the activation of PKA by the FSK treatment increased the reporter activity (Fig. 3e). RT-PCR analysis showed the FSK treatment slightly increased *Xbp1* splicing level in C3H10T1/2 cells (Supplementary Fig. 4b). However, the introduction of sXBP1-expression vectors into C3H10T1/2 cells never increased in the reporter activity, although s*Xbp1* mRNA level was higher than the FSK treatment (Fig. 3e and Supplementary Fig. 4b). Interestingly, the treatment with FSK of cells that were introduced with sXBP1-expression vectors showed the reporter activity more than FSK treatment alone (Fig. 3e). On the other hand, the introduction of vectors expressing a mutant sXBP1 lacking bZIP domain (ΔbZIP-sXBP1), which is not able to bind to DNA, never showed the reporter activity more than FSK treatment alone (Fig. 3e and Supplementary Fig. 4c). These data suggested that sXBP1 directly upregulates *Ucp1* by cooperating with other factors induced by the activation of PKA.

### The IRE1α-XBP1 pathway is activated in a PKA –dependent manner

**Figure 4:**
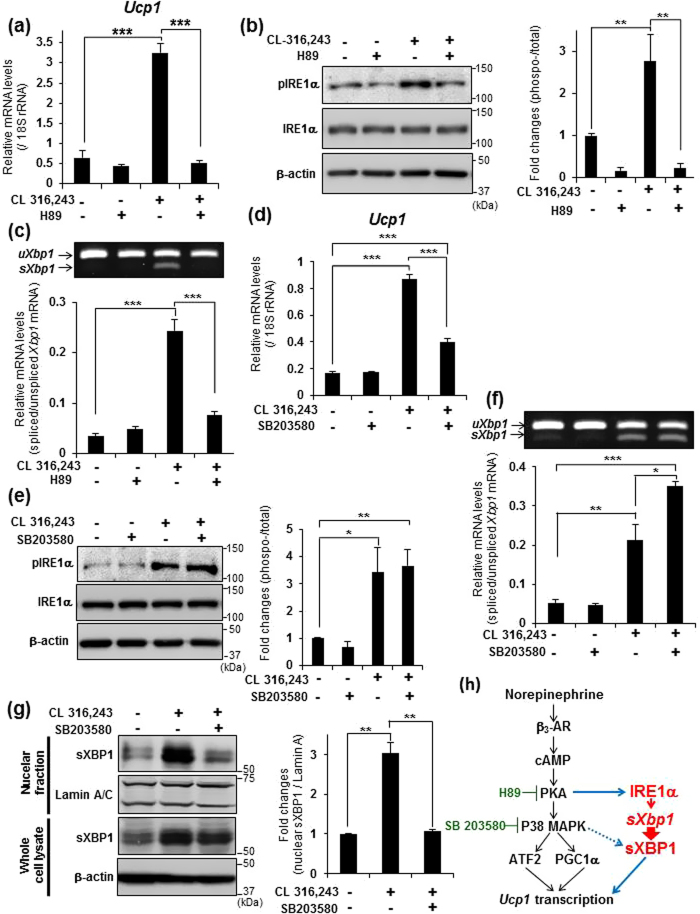
The IRE1α-XBP1 pathway is induced depending on PKA activities. (**a**) Real-time PCR analysis of *Ucp1* in brown adipocytes that were pre-treated with 40 μM H89 for 1 h, and then stimulated with 1 μM CL 316,243 for 3 h. (**b**) WB analysis of IRE1α in brown adipocytes that were pre-treated with 40 μM H89 for 1 h, and then stimulated with 1 μM CL 316,243 for 30 min. Graph on right shows the ratio of phosphorylated to total IRE1α. β-actin was used as a loading control. (**c**) RT-PCR analysis of *Xbp1* in brown adipocytes treated with H89 and CL 316,243 described as (**a**) (upper panel). Lower graph shows the quantification of *Xbp1* splicing levels. Data are mean ± S.D. (n = 4), **P < 0.01, ***P < 0.001. (**d**) Real-time PCR analysis of *Ucp1* in brown adipocytes that were pre-treated with 20 μM SB203580 for 30 min, and then stimulated with 1 μM CL 316,243 for 3 h. (**e**) WB analysis of IRE1α in brown adipocytes that were pre-treated with 20 μM SB203580 for 30 min, and then stimulated with 1 μM CL 316,243 for 30 min. Graph on right shows the ratio of phosphorylated to total IRE1α. β-actin was used as s loading control. (**f**) RT-PCR analysis of *Xbp1* in brown adipocytes treated with SB203580 and CL 316,243 described as (**d**) (upper panel). Lower graph shows the quantification of *Xbp1* splicing levels. Data are mean ± S.D. (n = 5), *P < 0.05, **P < 0.01, ***P < 0.001. (**g**) WB analysis of sXBP1 in the nuclear fraction or whole cell lysate of brown adipocytes that were pre-treated with 20 μM SB203580 for 1 h, and then treated with 1 μM CL 316,243 for 4 h. All samples were simultaneously treated with 10 μM MG132 for 4 h. Graph on right shows the amount of nuclear sXBP1. β-actin was used as loading control for whole cell lysates and Lamin A/C for the nuclear lysates. Data are mean ± S.D. (n = 3), **P < 0.01. (**h**) Our model of the transcriptional induction of *Ucp1* in brown adipocytes stimulated by norepinephrine.

The above findings led us to hypothesize that the IRE1α-XBP1 pathway is activated by the β_3_-AR pathway during the transcriptional induction of *Ucp1*. In the β_3_-AR signaling cascade, cAMP-dependent PKA activation leads to the phosphorylation of a p38 mitogen activated protein kinase (p38 MAPK). In turn, active p38 MAPK drives the transcriptional induction of *Ucp1* by the directly phosphorylating both ATF2 and PGC1α^32^. Because we found that IRE1α was heavily phosphorylated following FSK treatment, we first examined whether treatment with a PKA inhibitor H89 affects the IRE1α-XBP1 pathway. Treatment of primary brown adipocytes with 40 μM H89 suppressed the CL 316,243-induced expression of *Ucp1* (Fig. 4a). Interestingly, IRE1α phosphorylation and *Xbp1* mRNA splicing were also reduced drastically (Fig. 4b,c), indicating that activation of the IRE1α-XBP1 pathway is dependent on PKA activity. Next, to determine whether p38 MAPK regulates the activation of the IRE1α-XBP1 pathway, we treated primary brown adipocytes with a specific p38 MAPK inhibitor, SB203580. Treatment with 20 μM SB203580 significantly decreased the level of *Ucp1* mRNA (Fig. 4d). However, treatment with SB203580 failed to suppress IRE1α phosphorylation and *Xbp1* mRNA splicing (Fig. 4e,f). These findings were contrary to our expectations because inhibiting the IRE1α-XBP1 pathway significantly decreased the transcriptional induction of *Ucp1*. p38 MAPK has been reported to regulate the translocation of sXBP1 from the cytosol into the nucleus via phosphorylation^38^. Therefore, to determine whether SB203580 treatment inhibits the translocation of sXBP1 into the nucleus, we performed WB analysis using the nuclear fraction from brown adipocytes co-treated with CL 316,243 and SB203580. Co-treatment with CL 316,243 and SB203580 significantly reduced the amount of sXBP1 protein in the nucleus, which was increased by treatment with CL 316,243 (Fig. 4g).

Taken together, it was clear that the phosphorylation of IRE1α and the splicing of *Xbp1* mRNA occur in a PKA-dependent manner, and that the translocation of sXBP1 into the nucleus is regulated by p38 MAPK in brown adipocytes stimulated with norepinephrine.

## Discussion

Recent studies have suggested that signals mediated by the UPR are key factors that regulate lipid or glucose metabolism. Additionally, it has also been demonstrated that transgenic mice, in which UPR signaling was modulated, showed metabolic phenotypes, such as resistance to obesity^39^,^40^. In this study, we demonstrated that the IRE1α-XBP1 pathway is highly activated compared with the other UPR branches during the induction of *Ucp1* transcription in brown adipocytes, and its induction mechanism is independent of ER stress. Indeed, robust phosphorylation of IRE1α occurred within 30 min after treatment with CL 316,243 or FSK, which is not enough time for misfolded or unfolded proteins to accumulate in the ER lumen without treatment with ER stressors. The findings suggest that the β_3_-AR pathway activates the IRE1α-XBP1 pathway. Recently, it was reported that IRE1α in the liver is phosphorylated directly by PKA in response to glucagon^41^. The glucagon receptor is a G protein-coupled receptor that belongs to the same family as the β_3_-AR. The binding of glucagon to its receptor activates PKA in a cAMP-dependent manner. Additionally, treating hepatocytes with glucagon or epinephrine induced the phosphorylation of IRE1α, which was abrogated by co-treatment with H89. Actually, we observed that treatment with H89 greatly suppressed the phosphorylation of IRE1α in brown adipocytes. It is conceivable that the robust phosphorylation of IRE1α is mediated by PKA. Our study also showed that the inhibition of p38 MAPK suppressed the induction of *Ucp1* following treatment with CL 316,243, but it never suppressed IRE1α phosphorylation and *Xbp1* mRNA splicing, indicating that the activation of PKA induced by the binding of norepinephrine to the β_3_-AR causes the phosphorylation of IRE1α and the subsequent splicing of *Xbp1* mRNA. Interestingly, the amount of nuclear sXBP1 in brown adipocytes treated with SB203580 was reduced compared with that following CL 316,243 treatment. p38 MAPK has been reported to phosphorylate sXBP1 on amino acid residues Thr 48 and Ser 61, and the phosphorylation of these residues is crucial for the translocation of sXBP1 into the nucleus^38^. Taken together, we suggest that the β_3_-AR pathway has two roles: 1) it activates the phosphorylation of IRE1α and the splicing of *Xbp1* mRNA during the transcriptional induction of *Ucp1* downstream of PKA, and 2) it regulates the nuclear translocation of sXBP1 to promote its transcriptional activity downstream of p38 MAPK. Our model of the regulation of *Ucp1* transcription is summarized in Fig. 4h.

In the present study, the increase in the level of *sXbp1* mRNA after treatment with Tm never caused the upregulation of *Ucp1*. sXBP1 is a transcription factor that includes bZIP domain, and it binds to ERSEs and UPREs. We found sXBP1 binding sites, two UPRE-like and one ERSE-like sequences within 1-kb upstream of *Ucp1*, but the expression of sXBP1 never increased the reporter activity. It has been reported that bZIP transcription factors heterodimerize with other transcription factors through their bZIP domain to alter their binding sites, which means that a target gene is selected depending upon the combination of a counter transcription factors^42^,^43^,^44^,^45^. For example, ATF6 dimerizes with sXBP1 and binds to an UPRE, which ATF6 is not able to bind by itself^46^. Moreover, the ATF6-sXBP1 heterodimer possesses eight-fold higher binding affinity for the UPRE than the sXBP1 homodimer^46^. Indeed, we demonstrated that a combination of sXBP1 expression and FSK treatment enhanced the reporter activity, which was higher than that of the FSK treatment alone. These data implies that sXBP1 acts as a transcription factor for *Ucp1* by forming a heterodimer with a counterpart induced by the β_3_-AR pathway during thermogenesis in brown adipocytes. The failure of Tm to induce the transcription of *Ucp1* could be due to differences in the sets of transcription factors induced by the β_3_-AR pathway and ER stress. To elucidate this possibility, the identification of a counterpart of sXBP1 in the transcriptional induction of *Ucp1* and further experiments are needed.

We also found an increase in the expression of ER-resident chaperones in brown adipocytes stimulated by cold exposure and CL 316,243. Interestingly, the increase in expression of ER-resident chaperones persisted after treatment. In addition to the transcription of *Ucp1*, the uptake and utilization of lipids and glucose increases in brown adipocytes during thermogenesis^47^. Genes involved in glucose uptake, such as the glucose transporter, GLUT4, are especially upregulated in BAT exposed to cold^48^. Furthermore, the secretion of several proteins, for example, vascular endothelial growth factor (VEGF) and fibroblast growth factor 21 (FGF21), also increases in brown adipocytes stimulated by cold or other activators^34^,^49^. These cellular responses demand an increase in the folding capacity of the ER. It is possible that the transcription of ER-resident chaperones is promoted to process transmembrane and secretory proteins effectively in preparation for the impending acute protein load on the ER.

Here, we first suggested that the UPR is coupled to the transcriptional induction of *Ucp1* in brown adipocytes. Although it remains unclear how relevant and important the IRE1α-XBP1 pathway actually is in activating *Ucp1* transcription *in vivo*, our work provided a great possibility that the UPR plays a crucial role in brown adipocytes to upregulate *Ucp1*. An increase in *Ucp1* expression could raise the energy expenditure of the body, and prevent the accumulation of excess fat. Therefore, if a contribution of the IRE1α-XBP1 pathway to energy consumption *in vivo* is elucidated, the IRE1α-XBP1 pathway in brown adipocytes may have a potential for treating obesity.

## Materials and Methods

All the methods were carried out in accordance with the Hiroshima University guidelines for the care and use of laboratory animals. All of the experimental protocols were approved by the Committee of Animal Experimentation, Hiroshima University.

### Materials

CL 316,243, forskolin, H89, 4 μ8 C, thapsigargin, and tunicamycin, were purchased from Sigma-Aldrich. SB203580 was purchased from Calbiochem. MG132 was purchased from Wako. Treatment time was indicated in figure legends. Antibodies used in western-blotting analysis were as follows: anti-β-actin (A2228, Sigma-Aldrich), anti-phospho-eIF2α (#9721, Cell Signaling Technology), anti-eIF2α (#9722, Cell Signaling Technology), anti-IRE1α (#3294, Cell Signaling Technology), anti-sXBP1 (sc-7160, Santa Cruz Biotechnology), and anti-Lamin A/C (sc-6215, Santa Cruz Biotechnology). Anti-PERK (residues 1094–1114) and anti-ATF6 (amino acids 6–307) rabbit polyclonal antibodies were described previously^50^. Anti-phospho-IRE1α antibody was a gift from Dr. Urano (Washington University in St. Louis).

### Animals and Subcutaneous injection

Newborn mice and 8-week-old C57BL/6 male mice were used in this study.

CL 316,243 was dissolved in sterile saline, and injected subcutaneously into 8-week-old male mice. 24 hours after injection, we isolate mRNA from BAT and measured gene expressions.

### Cell isolation, culture, and differentiation

Brown pre-adipocytes were isolated from newborn wild-type mice by collagenase digestion as described previously^51^. The digested tissue was filtered through a 30-μm nylon screen (Miltenyi Biotec). Isolated cells were collected by the centrifugation (200 × g) for 5 min, seeded on 35-mm plate, and grown to confluence in differentiation medium (Dulbecco’s modified Eagle’s medium (DMEM) containing 10% fetal bovine serum supplemented with 20 nM insulin (Sigma-Aldrich), and 1 nM 3,3′,5-triiodo-l-thyronine [T3] (Sigma-Aldrich)). For the induction to mature brown adipocytes, preadipocytes were cultured for 48 h in differentiation medium further supplemented with 0.5 mM isobutylmethylxanthine (Sigma-Aldrich), 0.5 μM dexamethasone (Sigma-Aldrich), and 0.125 mM indomethacin (Sigma-Aldrich). After this induction period, the cells were maintained in differentiation medium for 4–5 days until exhibiting a fully differentiated phenotype with massive accumulation of multilocular lipid droplets. C3H10T1/2 cells were maintained in DMEM containing 10% fetal bovine serum.

### RNA isolation and Real-time PCR

Total RNA was isolated from BAT of 8-week-old male mice or brown adipocytes using ISOGEN (Wako) according to the manufacturer’s protocol. First-strand cDNA was synthesized in a 20 μl of reaction volume using a random primer (Takara) and Moloney murine leukemia virus reverse transcriptase (Invitrogen). Real-time PCR was performed in a 20 μl PCR reaction with KAPA SYBER FAST qPCR Kit (KAPA Biosystems) and primers at a concentration 0.5 μM each. PCR reactions were run in duplicate for each sample and quantified in Light Cycler 480 (Roche). Sequences of primers used in this study are listed in Supplementary Table.

### RT-PCR

The splicing of *Xbp1* was analyzed by performing PCR in a 20 μl reaction mix containing 0.5 μm of each primer, 0.2 mM dNTPs, 3 units of Taq polymerase, and 10× PCR buffer (Agilent). The PCR condition was as follows: 94 °C for 5 min; 25 cycles of 94 °C for 1 min, 60 °C for 1 min, and 72 °C for 1 min; and 72 °C for 7 min. The PCR products were resolved by electrophoresis on a 4.8% acrylamide gel. The density of each band was quantified using Image_analysis_software CS Analyzer 4 (ATTO CORPORATION). Sequences of primers used in this study are listed in Supplementary Table.

### Protein Extraction and Western-blotting analysis

Cells were lysed in 1% Triton buffer (20 mM HEPES, pH 7.5, 150 mM NaCl, 1% Triton X-100, 10% glycerol, 1 mM EDTA, 10 mM tetrasodium pyrophosphate, 100 mM NaF, 17.5 mM β-glycerophosphate, phosphatase inhibitor cocktail 3 (Sigma-Aldrich), and protease inhibitor cocktail set I (Wako)) for detection of phosphorylated or total PERK, eIF2α, and IRE1α. For detection of ATF6 and sXBP1 in whole cell lysates, the cells were lysed in hot-SDS buffer (0.9% SDS, 15 mM EDTA, and 8 mM methionine were boiled for 10 min, cooled, diluted to 0.3% SDS, and adjusted to contain 33 mM Tris acetate, pH 8.5, 1.7% Triton X-100, and protease inhibitor cocktail set I (Wako)). All samples were centrifuged at 4 °C for 15 min after incubation on ice for 45 min, and then the supernatants were collected. For nuclear protein extraction, cells were lysed in hypotonic buffer (20 mM Tris-HCl, pH7.4, 10 mM MgCl_2_, 1% Triton X-100, 10% Glycerol, 2 mM EDTA, 1 mM NaF, 2.5 mM β-glycerophosphate, 1 mM DTT, and protease inhibitor cocktail set I (Wako)). After incubation on ice for 15 min, samples were centrifuged for 10 min at 3,000 rpm and supernatant was discarded. Remaining pellet (Nuclear fraction) were lysed in hypertonic buffer (20 mM Tris-HCl, 500 mM NaCl, pH7.4, 10 mM MgCl_2_, 1% Triton X-100, 10% Glycerol, 2 mM EDTA, 1 mM NaF, 2.5 mM β-glycerophosphate, 1 mM DTT, and protease inhibitor cocktail set I (Wako)), and incubated on ice for 45 min. The supernatant was collected after centrifugation at 15,000 rpm for 15 min. Protein-equivalent samples were loaded onto SDS-polyacrylamide gels. The density of each band was quantified using Image_analysis_software CS Analyzer 4 (ATTO CORPORATION).

### Plasmids and Luciferase Assays

pcDNA3.1(+) vector expressing sXBP1 or ΔbZIP-sXBP1 were generated by PCR using primer set described in Supplementary Table. Luciferase reporter plasmid that contained the *Ucp1* promoter region (pUCP1-pro-Luc) was a gift from Dr. Kawada (Kyoto University). 200 ng pUCP1-pro-Luc reporters were co-transfected into C3H10T1/2 cells (3 × 10^4^ cells per well) in 24-well plates along with 20 ng Renilla luciferase (internal control) and 7.5 ng pcDNA3.1(+) vector expressing sXBP1 or ΔbZIP-sXBP1 using Screenfect A (Wako). 16 hours after the transfection, the transfected cells were treated with Forskolin for 8 hours. Cells were harvested into passive lysis buffer (Promega), and dual luciferase activity was assayed with a GloMax Multi+ Detection System (Promega). Reporter luciferase activity was normalized to the internal Renilla control activity.

### Statistical Analysis

Statistical comparisons were made using the unpaired Student’s t-test. Statistical significance between two samples was determined by a *p* value of less than 0.05. *p* values of less than 0.05, 0.01 or 0.001 are described as *p < 0.05; **p < 0.01; or ***p < 0.001, respectively.

## Additional Information

**How to cite this article**: Asada, R. *et al.* IRE1α-XBP1 is a novel branch in the transcriptional regulation of *Ucp1* in brown adipocytes. *Sci. Rep.* **5**, 16580; doi: 10.1038/srep16580 (2015).

## Supplementary Material

Supplementary Information
