# Supplementary material for: IRE1α-XBP1 is a novel branch in the transcriptional regulation of Ucp1 in brown adipocytes
Source: Sci Rep. 2015 Nov 16;5:16580. doi: 10.1038/srep16580 (PMC4644985; doi:10.1038/srep16580)
Supplement: Supplementary Information [file srep16580-s1.pdf]

IRE1α-XBP1 is a novel branch in the transcriptional regulation of *Ucp1* in brown adipocytes.

Supplementary Information

Rie Asada, Soshi Kanemoto, Koji Matsuhisa, Kenta Hino, Min Cui, Xiang Cui, Masayuki Kaneko, and Kazunori Imaizumi.

Supplementary Figure 1.

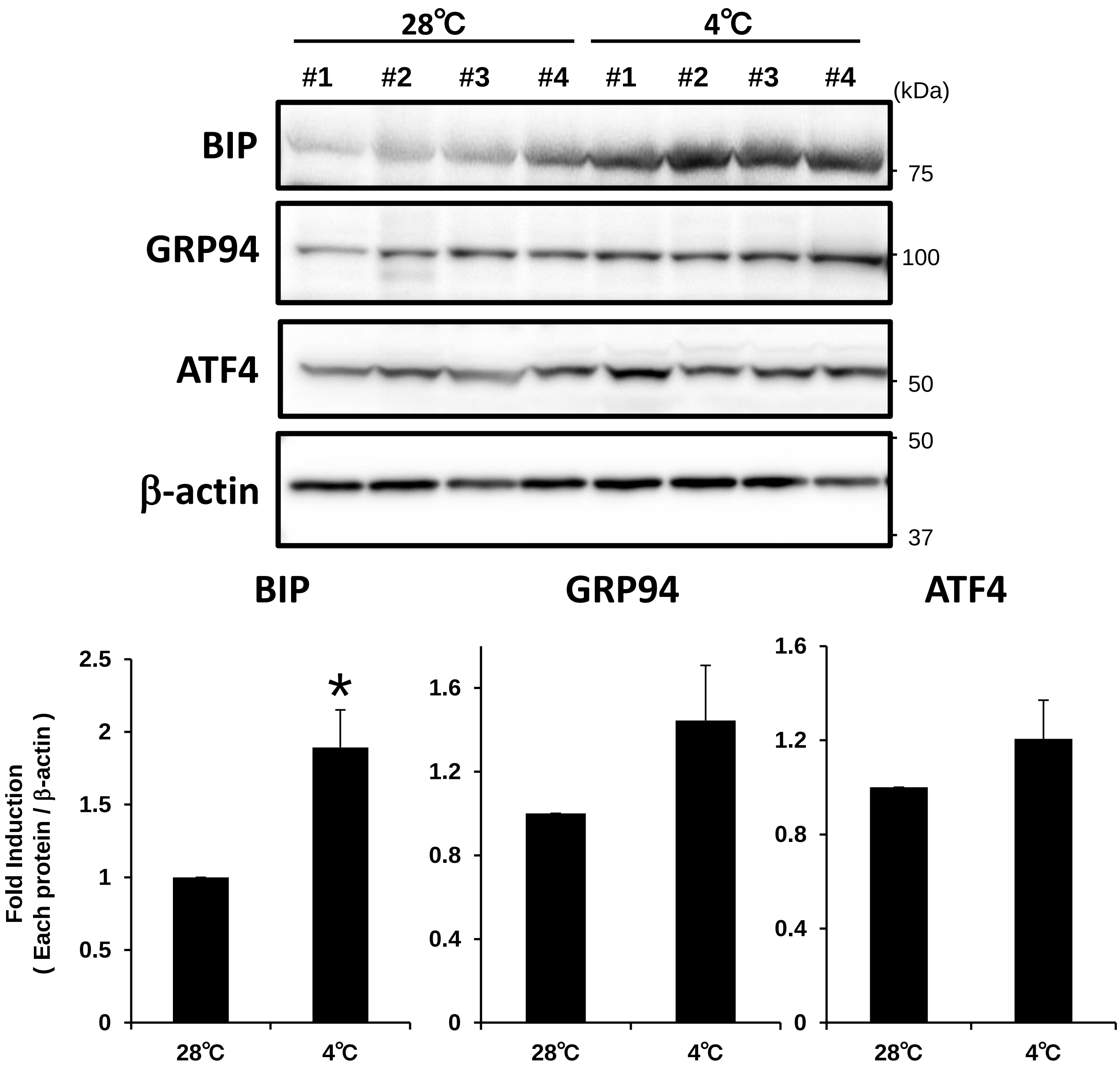

Supplementary Figure 1. Cold exposure increased UPR-related proteins in BAT.

Western -blotting (WB) analysis of Bip, GRP94, and ATF4 in BAT exposed to cold (4 ° C) for 24 h, or in control (28 ° C) BAT. β-actin was used as a loading control. Note that the significant increase in Bip protein was induced by cold exposure. Differences between control and cold exposure were analyzed by Student's t-test. Data are mean ±S.D. (control: n = 4, cold: n = 4) *P < 0.05.

Supplementary Figure 2.

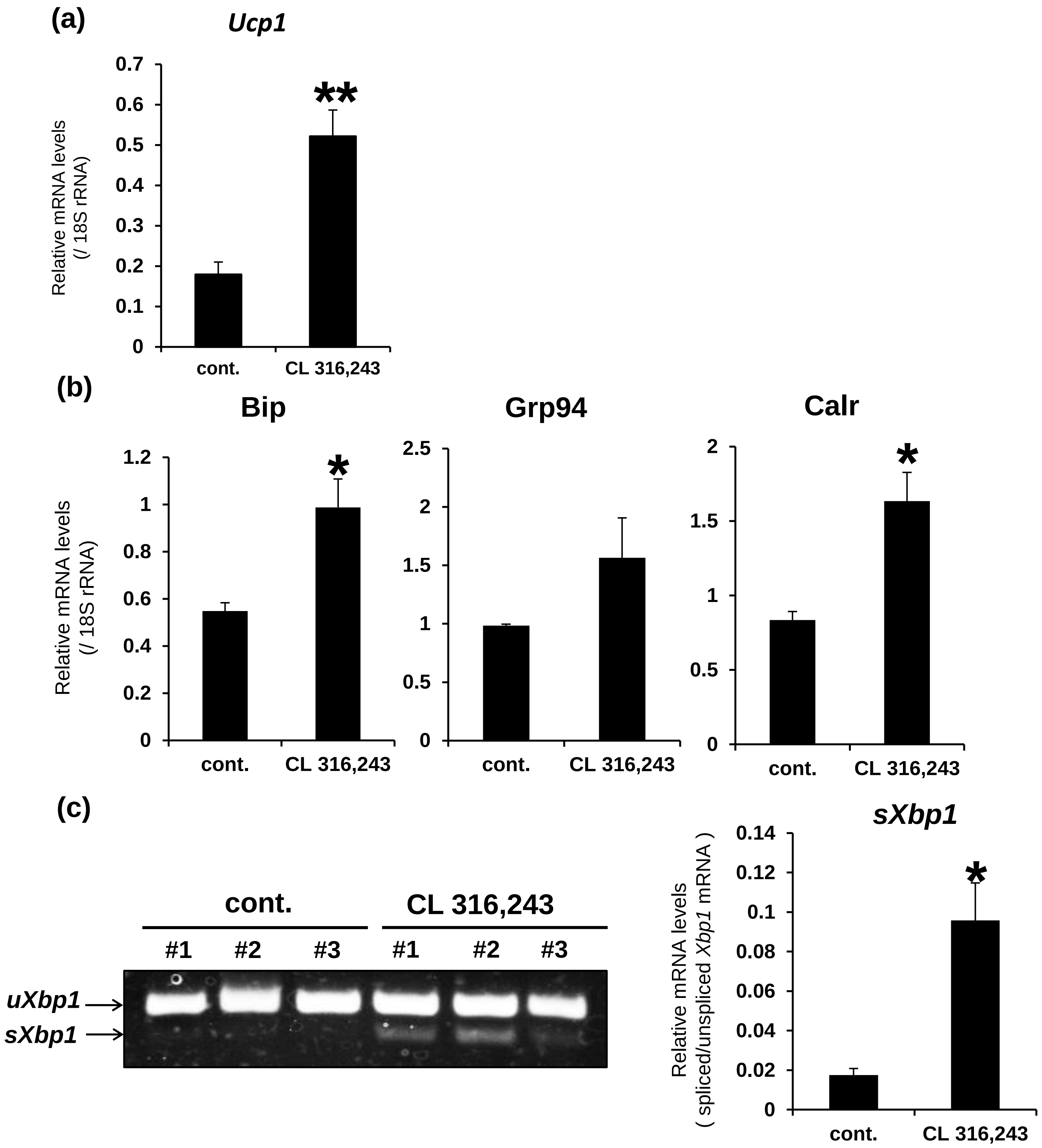

Supplementary Figure 2. UPR-related genes were upregulated by subcutaneous injection of CL 316,243 *in vivo*.

(a-b) Real-time PCR analysis of *Ucp1* (a), and UPR-related genes (b) in BAT of mice injected with CL 316,243. (c) RT-PCR analysis of *Xbp1* in BAT injected with CL 316,243 (left panel). *uXbp1* and *sXbp1* indicate unspliced and spliced forms of *Xbp1*, respectively. Graph on right shows the quantification of *Xbp1* splicing levels. Control mice were injected with sterile saline. Differences control and CL 316,243 were analyzed by Student's t-test. Data are mean ±S.D. (control: n = 3, CL 316,243: n=3), *P < 0.05.

Supplementary Figure 3.

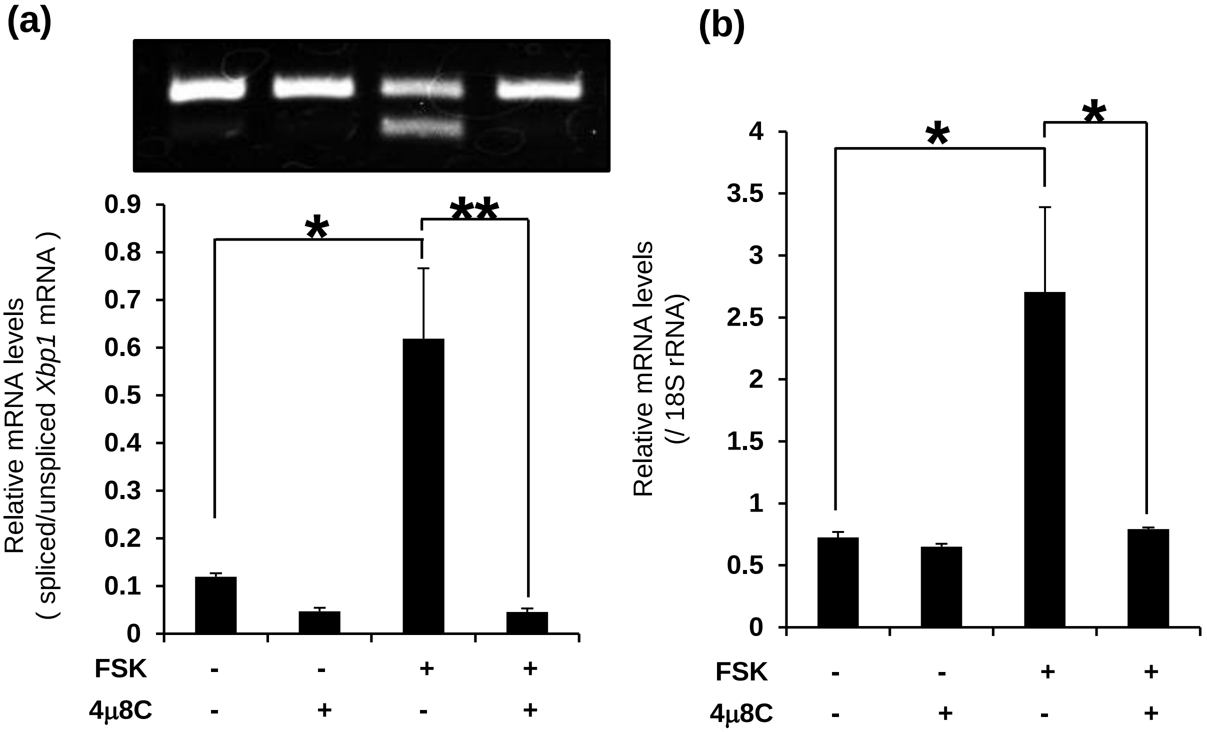

Supplementary Figure 3. Inhibition of the IRE1α-XBP1 pathway suppressed the increase in the *Ucp1* expression induced by forskolin.
(a) RT-PCR analysis of *Xbp1* in brown adipocytes that were pre-treated with 30 μM 4μ8C for 30 min and then stimulated with 20 μM forskolin (FSK) for 3 h (upper panel). Lower graph is the quantification of *Xbp1* splicing levels. (b) Real-time PCR analysis of *Ucp1* in brown adipocytes treated with 4μ8C and FSK described as (a). Note that treatment with 4μ8C significantly decreased *Ucp1* expression induced by FSK. Data are mean ±S.D. (n = 4), *P < 0.05, **P < 0.01.

Supplementary Figure 4.

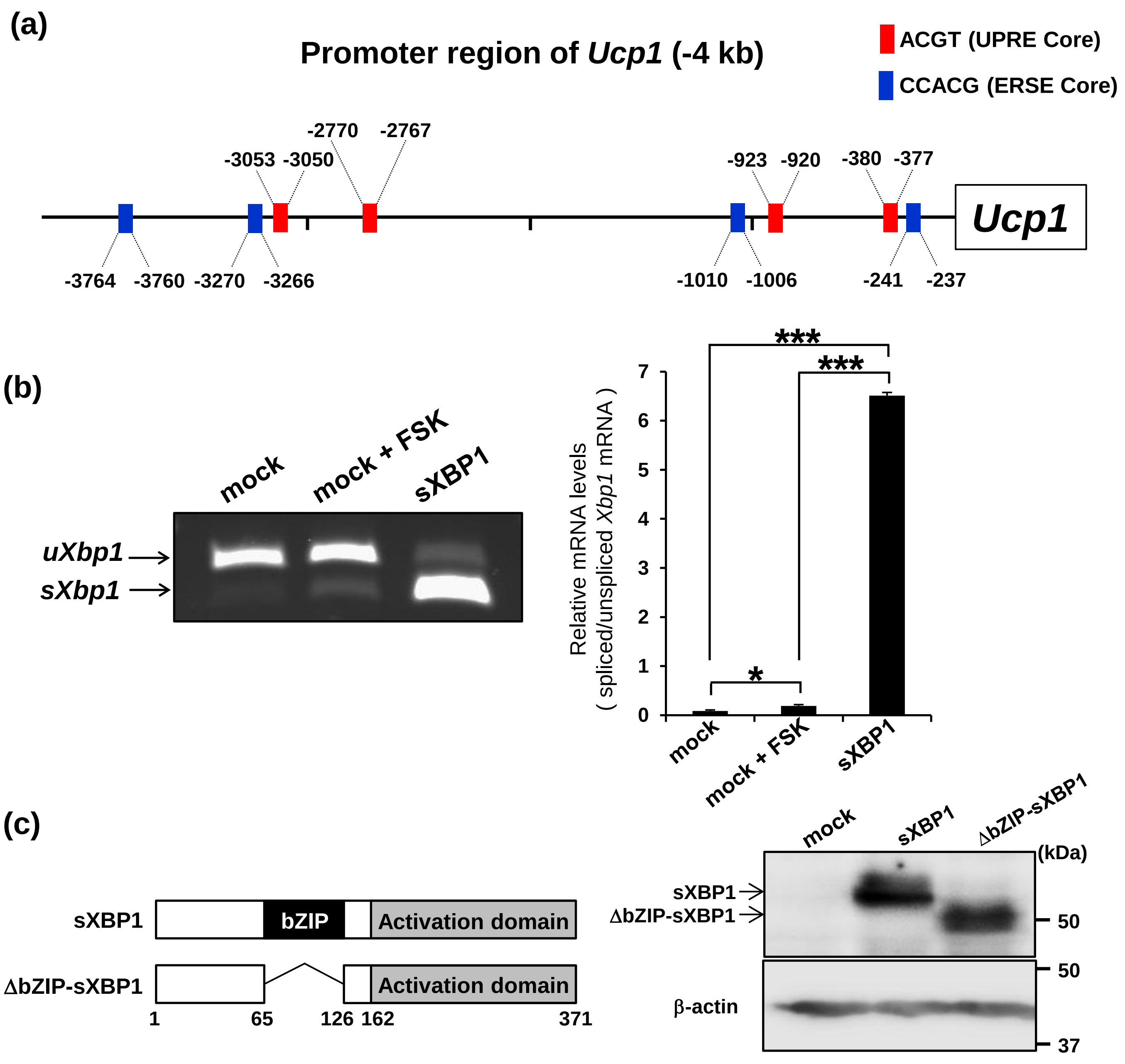

Supplementary Figure 4. A searching for sXBP1 binding sites, and RT-PCR or WB analysis of *Xbp1* in C3H10T1/2 cells.
(a) A schematic representation of sXBP1 binding sites in the promoter region of *Ucp1* (-4kb). Red boxes indicate UPRE core sequences and blue boxes indicate ERSE core sequences. Note that there are two UPRE and one ERSE core sequences within -1 kb region. (b) RT-PCR analysis of *Xbp1* in C3H10T1/2 cells transfected with mock or a vector expressing sXBP1 (left panel). *uXbp1* and *sXbp1* indicate unspliced and spliced forms of *Xbp1*, respectively. Graph on right shows the quantification of relative *sXbp1* mRNA levels. forskolin (FSK) treatment time was 4 h. Data are mean ±S.D. (n = 3), *P < 0.05, ***P < 0.001. (c) A schematic representation of sXBP1 and ΔbZIP-sXBP1 constructs (left panel). The WB analysis of C3H10T1/2 cells transfected with vectors expressing sXBP1 or ΔbZIP-sXBP1 (right panel). The experiment was independently reproduced three times.

Supplementary Table

Primer sets used for Real-time PCR or RT-PCR analysis		
Gene		Sequence
Ucp1	Fwd	caaatcagctttgcctcactc
	Rev	acaagctttctgtggtggcta
Bip	Fwd	gtttgctgaggaagacaaaaagctc
	Rev	cacttccatagagtttgctgataat
Grp94	Fwd	tgtgcagagagaggaagaagc
	Rev	aactcctcatttccagcgagt
Calreticulin	Fwd	tgatgctaagaagcctgagga
	Rev	tctaggcccagtacagcaaaa
Xbp1	Fwd	acacgcttgggaatggacac
	Rev	ccatgggaagatgttctggg
Erdj4	Fwd	ccatgaagtaccaccctgaca
	Rev	gaccgagtgttttggttctga
18S rRNA	Fwd	tggataccgcagctaggaata
	Rev	tcgtttatggtcggaactacg
**Primer set used for construction PCR**		
Construct		Sequence
sXBP1	Fwd	caccatggtggtggtggcagcggc
	Rev	ttagacactaatcagctggg
ΔbZIP-sXBP1	Fwd	agaacacgcttgggaatggacac
	Rev	ctcctccgggctcaggtgcgtgagc
